# Good practice recommendations on implementation evaluation for policies targeting diet, physical activity, and sedentary behaviour

**DOI:** 10.1186/s12889-023-15775-9

**Published:** 2023-06-28

**Authors:** Janine Wendt, Daniel A. Scheller, Anna Banik, Aleksandra Luszczynska, Sarah Forberger, Hajo Zeeb, Marie Scheidmeir, Thomas Kubiak, Nanna Lien, Biljana Meshkovska, Karolina Lobczowska, Piotr Romaniuk, Agnieszka Neumann-Podczaska, Katarzyna Wieczorowska-Tobis, Jürgen M. Steinacker, Annabel S. Mueller-Stierlin

**Affiliations:** 1 Department of Internal Medicine, Division of Sports and Rehabilitation Medicine, University Hospital Ulm, Ulm, Germany; 2 Department of Psychology, SWPS University of Social Sciences and Humanities, Wroclaw, Poland; 3 Leibniz-Institute for Prevention Research and Epidemiology-BIPS, Bremen, Germany; 4 Health Psychology, Johannes Gutenberg University, Mainz, Germany; 5 Department of Nutrition, University of Oslo, Oslo, Norway; 6 Department of Health Policy, Medical University of Silesia, Katowice, Poland; 7 Department of Palliative Medicine, Poznan University of Medical Sciences, Poznan, Poland; 8 Department of Psychiatry & Psychotherapy II, Ulm University, Günzburg, Germany; 9 Institute for Epidemiology & Medical Biometry, Ulm University, Ulm, Germany

**Keywords:** Policy, Implementation, Evaluation, Health promotion, Lifestyle

## Abstract

Health policies aim to achieve specific health goals through system-level changes, unlike common health interventions that focus on promoting specific health behaviors on individual level. However, reliable data on the feasibility and implementation of policy actions across Europe are lacking. Moreover, no practice-oriented guidance exists for policy makers and implementers on how to evaluate policy implementation.

As part of the Policy Evaluation Network, we aimed to synthesise knowledge on how to evaluate the implementation of policies promoting healthy diets, physical activity, and reducing sedentary behaviours. The multidisciplinary working group comprised 16 researchers and conducted two scoping reviews, three systematic reviews, two meta-reviews, two qualitative case studies and one quantitative case study over three years. The target populations included the general population, those at risk for obesity, and school children. Based on these reviews and case studies, this article summarises and presents the findings and lessons learned regarding the implementation evaluation of policies in nine case reports.

Drawing on these experiences, three critical requirements for policy implementation evaluation were set: 1) conduct a comprehensive policy implementation evaluation from a multi-level perspective, 2) use implementation frameworks to address processes, determinants, and outcomes, and 3) engage relevant stakeholders in policy implementation evaluation. Finally, the consensus process resulted in 10 steps for the implementation evaluation of policies to promote physical activity and a healthy diet and to reduce sedentary behaviours, which adhere to the requirements and resources of the targeted policy.

The findings of an implementation evaluation can lead to a better understanding of why policies work or not and can serve as a basis for developing solutions. This practice-oriented guidance outlines factors that should be considered in policy implementation evaluation to address its complexity. In this way, involved researchers and practitioners are empowered to engage in the evaluation process to close the knowledge gap regarding policy implementation.

**Supplementary Information:**

The online version contains supplementary material available at 10.1186/s12889-023-15775-9.

## Background

Recognizing that a healthy lifestyle is the most effective way to minimize the risk of developing non-communicable diseases [1], the number of (public) health policies to promote physical activity and healthy diet and to reduce sedentary behaviours has been growing in recent years [2, 3]. Policies are “purposeful decisions, plans and actions made by voluntary or authoritative actors in a system designed to create system-level change to directly or indirectly achieve specific societal goals. Within this definition, public policy is a form of government action usually expressed in a law, a regulation, or an order as it reflects an intent of government or its representative entities” [4–6]. In contrast to interventions, the role of policies is to change systems, not individuals. In this respect, they provide a framework in which interventions are tendered, developed, financed or implemented [7].

It is now well recognised that health policies do not succeed or fail on their own merits but rather due to challenges of certain steps in the policy process [8], including those during implementation [9]. According to the policy process, policy implementation occurs when the policy is translated into operational practice. The implementation process takes place in different stages (e.g., 1) exploration, 2) adoption decision/preparation, 3) active implementation, 4) sustainment [10]) and is realised by means of implementation strategies (also referred to as policy instruments) such as training for implementers, formal practice protocols and guidelines as well as economic, fiscal, and regulatory strategies [11]. The policy implementation process usually involves top-down strategies (a centralised approach starting from a central body such as the government) and bottom-up strategies (a decentralised approach involving implementers and stakeholders). In addition to the implementation strategies and the implementation actors involved, the target groups and the policy's characteristics are constantly interacting with a broad cultural, social, economic and political context [12, 13]. This implies that a broad variety of factors can influence the implementation process. These factors are also described as determinants or dichotomised according to their effect on the process in barriers, obstacles, impediments, enablers and facilitators in implementation science [14]. Implementation outcomes refer to the effects or results of the implementation process e.g. how well a policy is implemented in practice and how it impacts the target population and other stakeholders. They are indicators of implementation success and key intermediate outcomes related to policy outcomes (e.g., changes in behaviour, awareness, attitudes, or knowledge) and health outcomes (e.g., increased physical fitness) [4, 15]. The implementation of a policy can be considered successful if it creates a supportive context to reduce the risks of developing non-communicable diseases and empower individuals to adopt and maintain healthy behaviours. Extensive planning and monitoring of policy implementation is needed to optimize the implementation process and maximize policy and health outcomes. In contrast to the evaluation of the impact on health outcomes, which usually takes place at a later stage after implementation, implementation evaluation should occur throughout the implementation process. In this way, implementation determinants can be identified and implementation strategies adjusted in a timely manner given the dynamic context in which policies are implemented. In addition, the implementation evaluation should also aim to assess the influence of attitudes, knowledge and awareness of actors and stakeholders regarding the changes. Policy implementation evaluation aims “to understand how the policy was translated in operational practice and to identify the occurrence and variation of intended and unintended outcomes” [8]. Ultimately, the results of the implementation evaluation can serve as a basis for identifying and implementing solutions [8].

Until recently, there was hardly any knowledge about implementing and evaluating policies targeting dietary behaviour, physical activity, and sedentary behaviour across Europe [16–18]. Therefore, since 2019, the Policy Evaluation Network (PEN) has been collaborating to advance the evidence base on the effective implementation of such policies and their impact in terms of improving health behaviours [5, 19]. A subgroup of PEN partners has focused on key requirements of policy implementation evaluation by identifying 1) key aspects of implementation processes, 2) key facilitators and barriers of policy implementation, and 3) tools to assess implementation processes, facilitators, and barriers related to policies promoting a healthy diet and physical activity. To provide practice-oriented guidance on the evaluation of policy implementation for researchers and further actors involved, this paper summarizes the findings and experiences of this working group. Given the complexity of the topic and the limited empirical evidence, we do not aim to provide an evidence-based guideline, but an outline of the approach that could be used to develop further respective guidelines for the implementation evaluation of healthy diet and physical activity policies.

## Methods

### Development process of the practice-oriented guidance for policy implementation evaluation

The multidisciplinary working group comprised 16 researchers with expertise in implementation science, epidemiology, political science and health science, including the disciplines of nutrition, exercise, medicine, and health promotion. Between 2019 and 2022, seven reviews and three case studies were conducted to identify key requirements of policy implementation evaluation (see Supplementary Table). Our experiences and findings of this research are summarized in case reports, including their objectives, methods, findings & lessons learned:

- case report 1: scoping reviews on implementation processes of sugar-sweetened beverage taxation and public physical activity policies [20, 21]
- case report 2: a qualitative study on implementation determinants of the EU School Fruit and Vegetables Scheme [under review]
- case report 3: a meta-review on social, economic, political, and geographical context determinants for policies promoting a healthy diet and physical activity [22]
- case report 4: a systematic review of frameworks for implementation of policies promoting a healthy diet and physical activity [23]
- case report 5: a systematic review on acceptability of policies targeting dietary behaviours and physical activity [24]
- case report 6: meta-review of implementation determinants for policies promoting a healthy diet and physical activity [25]
- case report 7: a systematic review on implementation determinants of direct fruit and vegetables provision interventions in kindergartens and schools [26]
- case report 8: a quantitative cross-sectional study on determinants for the adoption of physical activity policies in elementary schools [27]
- case report 9: participatory stakeholder studies on implementation determinants following the Theory of Change approach [28]

Consequently, the findings and experiences gained in conducting these reviews and case studies were integrated into this practice-oriented guidance for policy implementation evaluation following a modified Delphi approach [29, 30] that didn’t rely on a series of questionnaires only, but also on subsequent meetings and revised listings of recommendations: In doing so, the evidence and experience with policy implementation evaluation gained by the working group over the past three years served as a starting point. Three authors extracted key lessons from internal work package reports and manuscripts (AMS, JW and JS). Subsequently, a survey was conducted in November 2021 that asked the working group to provide their opinion on the relevance and meaning of each key lesson. Successive feedback and further input were gathered from all working group members through two virtual workshops, including polls, held in December 2021 and March 2022. Revisions included changes to provisional recommendations, the addition of further recommendations and the integration of other scientific literature as suggested by members of the working group. The final guidance was checked and agreed upon by all working group members.

## Results

### Practice-oriented guidance for policy implementation evaluation

Through intense exchanges and discussions, the working group identified four themes of particular importance in the policy implementation evaluation of policies promoting a healthy diet or physical activity. The case reports were then assigned to these themes to illustrate real-life experiences in the field. The key themes were 1) scarcity of policy implementation evaluation (Case report 1), 2) complexity of policy implementation evaluation (Case reports 2 and 3), 3) frameworks for policy implementation evaluation (Case reports 4, 5, 6 and 7), and 4) participatory approaches for policy implementation evaluation (see Case reports 1, 8, and 9). The following subsections provide detailed descriptions of the results we obtained for each of the above mentioned thematic areas.

Finally, as a result of the Delphi process, the implications of these experiences were expressed as three key requirements for policy implementation evaluation. In addition, to increase practice-orientation, the working group decided during the Delphi-process to incorporate their experiences in conducting these studies in a 10-step instruction based on work by Public Health Ontario [31] and the Melbourne School of Population and Global Health [32], respectively.

#### Evidence of policy implementation evaluation is scarce

While awareness of the need for implementation evaluation of health related policies is increasing and work has already been done in this area, the implementation of policies in the field of nutrition and physical activity is still rarely evaluated [16–18]. Furthermore, hardly any research covers implementation and its evaluation from a comparative perspective across Europe [5, 19]. Therefore, we prepared two scoping reviews depicting the implementation processes of sugar-sweetened beverage (SSB) taxation and public physical activity (PA) policies. The focus of the work was based on the priorities within the PEN project (taxation of SBB and promotion of PA) [5]. In the end, only a few implementation cases (SSB: 6, PA: 10) could be identified. The transferability of identified implementation processes between the limited number of settings was very poor (see Case report 1).

#### Case report 1: what do we know about the actual implementation process of sugar-sweetened beverage taxation and public physical activity policies: results from two scoping reviews

##### Objective

(1) To examine implementation processes for sugar-sweetened beverage (SSB) taxation in terms of (i) pre-implementation context, (ii) taxation instruments used and (iii) interactions in the implementation process and (2) to examine implementation processes for policies targeting physical activity (PA) in terms of (i) the policies covered and their legal quality, (ii) the actors and stakeholders involved in the implementation process and (iii) the used implementation strategies (vertical: incorporating core policy elements within one policy area into the next level of government and administration (e.g., from national state to federal state governments); horizontal: the mainstreaming of core policy elements into other policy areas on one level of the government (e.g., from transportation to education); or a mix).

##### Methods

Scoping reviews were systematically conducted (registered Open Science Framework: osf.io/7w84q/), searching ten databases and grey literature for SSB until February 2020 and PA until March 2022. For SSB and PA 1,248 and 7,741 titles and abstracts were screened, respectively. The selection of variables to be extracted was based on the policy cycle stages (agenda setting, formulation, adoption, implementation, monitoring & evaluation) and informed by intervention implementation research.

##### Findings & lessons learned

Only six implementation cases based on three publications could be identified for SSB taxation. SSB taxation was implemented by hiring a subcontractor or using pre-existing tax collection structures. Political and public support within the implementation process was reported in only two cases. Regarding public PA policies, ten publications could be included for analysis. Education (school sector) and the promotion of PA in general (national PA plans or citywide approaches) were the identified policy areas. The legal classification included laws (school sector), coordination and budgeting, and non-legally binding recommendations and the jurisdictions were federal (*n* = 4), state (*n* = 1), county (*n* = 1), school district (*n* = 1), and city (*n* = 3). The following implementation strategies were identified: (1) citywide approaches: coordinated approach with vertical and horizontal integration; (2) federal PA policies: mix of implementation strategies; (3) school sector: strict horizontal top-down integration without the involvement of other actors. The publications mostly covered high-income countries, were highly diverse, fragmented and did not allow for generalisations. The reporting standards and topics reported were also different in all papers. This highlights that data on policy implementation is scarce. Further research on policy implementation processes is urgently needed to identify the most effective strategies and close this knowledge gap. Further, reporting standards must be developed and implemented to allow comparisons.

For further details, see [20, 21]

### Policy implementation evaluation is complex and requires specialized methods

When evaluating policy implementation, it is essential to bear in mind that the policy and the context in which it is embedded are highly complex. On the one hand, the policy to be implemented is the product of what has happened in earlier stages of the policy process. On the other hand, the individual actions taken by different actors (e.g., political decision-makers, implementers and stakeholders) related to this policy “may be substantially modified, elaborated or even negated during the implementation stage” [33] in response to implementation determinants. Moreover, policy implementation is driven by multiple actors working in multidisciplinary networks and using various methods. Consequently, policy implementation varies according to the context in which the policy is implemented. These aspects of policy implementation have to do with power and social interaction and can hamper the prediction of policy outcomes and impact on health outcomes. In summary, policy implementation is highly complex (see Case reports 2 and 3), so special evaluation methods are needed. These do not necessarily comply with the standards of medical sciences (such as process evaluation embedded in randomized controlled trials or predictive modelling) but instead have to consider multiple perspectives in a participatory approach.

#### Case report 2: barriers and facilitators to implementation of the EU school fruit and vegetables scheme: cross country study using the Consolidated Framework for Implementation Research

##### Objective

To identify barriers and facilitators to the implementation of the EU School Fruit and Vegetables Scheme from the perspective of country-level government implementers.

##### Methods

Data collection and analysis of this qualitative, explorative study was guided by the Consolidated Framework for Implementation Research (CFIR) [34]. In total, 23 semi-structured interviews were conducted with 29 respondents from 10 EU countries and at the EU level. Among them were representatives from ministries of agriculture, health, and education. Qualitative data was coded inductively, and the codes were assigned to constructs of the CFIR.

##### Findings & lessons learned

 Content related to 19 out of 26 constructs of the CFIR could be identified. Some example constructs are: adaptability, external policy and incentives, networks and communications, knowledge and beliefs and executing. The same influencing factors may be perceived either as barriers or facilitators depending on the level of implementation we are looking at. Namely, what may be a facilitator at the political level, may be a barrier at the public administration level and a barrier further down in the school setting. Thus, when evaluating (public) policy derived programmes where governments are involved in implementation, theories from the policy field could usefully complement the implementation science theories and models [35]. For example, Matland’s Ambiguity-Conflict Model [36] explains how ambiguity and conflict at different levels affect policy implementation.

#### Case report 3: social, economic, political, and geographical context that counts: meta-review of implementation determinants for policies promoting healthy diet and physical activity

##### Objective

To synthesize the evidence of the context-related implementation determinants of policies targeting physical activity, sedentary behaviour, or healthy diet in the general population or populations at risk of obesity and in specific settings (school, workplace).

##### Methods

Based on a systematic search of nine databases and documentation of nine major stakeholders until February 2020, a systematic review of published reviews and stakeholder documents (evidence-based policy guidelines at the national or international level, from governmental and non-governmental organisations) was conducted. The titles and abstracts of 3,774 peer reviewed articles and 52,961 stakeholder documents were screened. Potential context-related determinants were assigned to the Context and Implementation of Complex Interventions (CICI) framework [12] and coded as belonging to the macro-level (national or country level) and meso/micro-level (community/organizational or individual level) (PROSPERO, #CRD42019133341).

##### Findings & lessons learned

A total of k = 25 reviews and k = 17 stakeholder documents were included. Overall, macro-level economic and political context-related determinants were found to be relevant for implementing these policies (e.g. availability of funds to support sustainable implementation and national/regional policies already operating in the setting). Furthermore, political, economic, and socio-cultural determinants at the meso/micro level were identified. The contexts might both enhance and hamper implementation processes. As the contexts can change over time and not all determinants can be anticipated before policies are implemented, constant monitoring should be carried out to adjust implementation strategies if necessary.

Using the original description of CICI domains, which provides a relatively general operationalisation illustrated with specific examples, has made capturing meso-level determinants, such as organizational culture, challenging. Furthermore, as the CICI framework differentiates between implementation strategies and context, the behaviours of implementers (e.g., staff support for implementation) were not captured as context-related factors.

For further details, see [22].

#### Policy implementation evaluation benefits from fitting frameworks

The use of implementation science theories, models, and frameworks (TMFs) is advisable to improve the comparability of implementation evaluation results across studies. According to Nilsen, their use aims to 1) describe and/or guide the process of translating research into practice, 2) understand and/or explain what influences implementation outcomes, and 3) evaluate implementation [35]. A large number of implementation science TMFs that differ in terms of purpose, content, or applicability to different contexts exists [35, 37, 38]. Therefore, especially for actors with limited experience in the field of implementation science, it can be a challenge to choose an appropriate approach depending on the research question.

Given the myriad of available implementation TMFs from various disciplines, we have focused on the applicability of frameworks in policy implementation evaluation within several systematic reviews (see Case reports 2, 4, 5, 6 and 7). The choice of a TMF for policy implementation evaluation must occur given the behaviour the policy wants to address (e.g., diet or physical activity) and the type of evaluation (e.g., monitoring of implementation process or determinants to adjust implementation strategies). Special consideration should be given to the context (e.g., political and/or socio-cultural) in which the policy under study is embedded. For this purpose, a combination of several TMFs or further adaptations of the TMF of choice might be necessary. If successfully chosen, the TMF will help to focus on the essential implementation processes, outcomes, or determinants in the complexity of policy implementation evaluation. However, most implementation science TMFs have not been developed for or tested in policy implementation. Compared to TMFs developed to guide interventions, policy implementation frameworks may have their specificity, for example, regarding the role of the political context and the institutions involved [39]. Our recent systematic review provides a summary of frameworks for the implementation of policies promoting healthy nutrition and a physically active lifestyle according to their scope, the content of the included constructs, the relationships between the constructs, and the inclusion of equity factors (see Case report 4) [40]. In general, the interplay between contextual factors and equity factors has not been adequately addressed so far. Moreover, it is necessary to know more about how to combine different TMFs while preserving the theoretical constructs and assumptions. Therefore, further guidance that outlines how to select and adapt implementation TMFs according to the policy to be examined and the respective evaluation questions should be provided.

In light of the high importance of TMFs in policy implementation evaluation, we have conducted four reviews on this matter (Case reports 4, 5, 6 and 7). Case reports 6 and 7 apply one of the most frequently used implementation frameworks [41], the Consolidated Framework for Implementation Research (CFIR) [34]. The framework offers an overarching typology including 26 key determinants, which are grouped into the following five domains: intervention characteristics, outer setting, inner setting, characteristics of individuals, and process [34].

#### Case report 4: frameworks for implementation of policies promoting healthy nutrition and physically active lifestyle: systematic review

##### Objective

To provide an overarching synthesis of frameworks guiding the implementation of healthy nutrition, physical activity, and sedentary behaviour policies.

##### Methods

Following a systematic search in nine research databases and eight stakeholder websites (e.g., The National Institute for Health and Care Excellence (NICE; United Kingdom), European Commission, World Health Organization-Regional Office for Europe (WHO)) until February 2020 (CRD42019133251), titles and abstracts of 1,578 of peer reviewed articles and 147,887 stakeholder documents were screened. The Data were coded according to 5 categories: (1) scope of content, (2) level of constructs, (3) types of relationships between constructs, (4) equity factors, (5) direct focus on particular behaviour (e.g., nutrition) vs. discussed application for particular behaviour.

##### Findings & lessons learned

In total, 38 policy implementation frameworks were reviewed. Frameworks focusing on policy implementation vary in terms of their scope, the content of included constructs (e.g., referring to implementation processes, determinants, or implementation evaluation), the level at which these constructs operate (e.g., the individual level, the organizational/community level), the relationships between the constructs, and the inclusion of equity factors. The findings of this review may facilitate the selection of a framework that best meets the needs and aims of each policy implementation evaluation. While most frameworks (55%) did not account for any of the investigated equity factors (gender, age, economic status/education/literacy, ethnicity, geographic isolation/distance, and culture), four identified frameworks [12, 42–44] accounted for all six equity factors. To address the political and ethical requirement for health equity, it is important to consider these equity factors in the evaluation. Choosing one of these frameworks can thus be recommended. Alternatively, it is possible to complement a chosen framework with equity factors.

For further details, see [23].

#### Case report 5: acceptability of policies targeting dietary behaviours and physical activity: a systematic review of tools and outcomes

##### Objective

To identify tools used to assess the acceptability of policies targeting physical activity and dietary behaviour, and to examine if acceptability differs depending on the policy's and respondents' characteristics.

##### Methods

A systematic review of public support for health policies with a focus on physical activity and dietary behaviour was updated (studies published in English between 2010 and 2021) searching three databases (PROSPERO, CRD42021232326). A total of 7,236 titles and abstracts were screened. Five categories of information were coded and extracted: (1) types of measures used to gain information on acceptability, (2) levels of acceptability, (3) characteristics of target behaviour, (4) characteristics of policies, (5) characteristics of target respondents: age, sex, country and socioeconomic status.

##### Findings & lessons learned

Only three of 48 included studies provided a theoretical foundation for their acceptability assessment. Studies using validated tools and a theoretical foundation to monitor acceptability are urgently needed to examine opportunities to increase the acceptability of policies. In this way, it is advisable to follow Proctor’s taxonomy [15] that provides eight conceptually distinct implementation outcomes along with their nominal definitions: acceptability, adoption, appropriateness, feasibility, fidelity, implementation cost, penetration, and sustainability. Acceptability is distinguished from service satisfaction and is defined as “the perception among implementation stakeholders that a treatment, service, practice, or innovation is agreeable, palatable, or satisfactory” [15]. This framework was precious in the screening process, as it facilitated coordination among reviewers to delineate acceptability from other implementation outcomes.

For further details, see [24].

#### Case report 6: meta-review of implementation determinants for policies promoting healthy diet and physically active lifestyle: application of the Consolidated Framework for Implementation Research

##### Objective

To identify determinants that may affect the implementation processes of policies targeting healthy diet, promoting physical activity, and/or reducing sedentary behaviour.

##### Methods

A meta-review of published systematic scoping or realist reviews and stakeholder documents was conducted. Data from nine databases and documentation of nine major stakeholders (e.g., The National Institute for Health and Care Excellence (NICE; United Kingdom), European Commission, World Health Organization—Regional Office for Europe (WHO)) were systematically searched until February 2020. A total of 3,774 titles and abstracts of peer reviewed articles and 52,961 stakeholder documents were screened. Data were coded according to 5 categories: (1) policy, (2) implementation, (3) healthy diet policy, (4) physical activity/sedentary behaviour policy, (5) school setting policies. Potential determinants were categorized using the Consolidated Framework for Implementation Research (CFIR) (PROSPERO, #CRD42019133341).

##### Findings & lessons learned

A total of k = 25 reviews and k = 17 stakeholder documents were included. We have identified the following seven CFIR-based implementation determinants among four domains: cost (domain: policy characteristic); networking with other organizations/communities, external policies (domain: outer setting); structural characteristics, implementation climate, readiness for implementation (domain: inner setting); knowledge/beliefs of involved individuals (domain: individual characteristics). While published reviews provided strong support for determinants of the inner setting and individual characteristics domains, stakeholder documents supported determinants of the outer and inner setting domains. When considering the content of the outer setting determinants listed in the stakeholder documents, these refer to, e.g., inter-sectoral collaboration, co-occurring governmental regulations, national and local policies, characteristics of legal regulations, and funding schemes. These implementation determinants were not well reflected in the CFIR. However, new constructs such as “local conditions” and “financing” have been defined in the recently updated version of the CFIR [45]. Whether these adjustments are sufficient to apply the CFIR in evaluating policy implementation remains to be investigated. Overall, the findings of this meta-review indicate that the identified determinants should be prioritized (if needed) when planning and monitoring the implementation of policies. For further details, see [25]

#### Case report 7: barriers and facilitators to implementation of direct fruit and vegetables provision interventions in kindergartens and schools: a qualitative systematic review applying the consolidated framework for implementation research (CFIR)

##### Objective

To systematically review qualitative results reporting on the determinants (barriers and facilitators) of implementing interventions that directly provide fruit and vegetables in kindergarten and school settings.

##### Methods

Following a systematic search of original research articles in six databases until July 2020, the titles and abstracts of 5,427 articles were screened. Identified determinants were assigned to the constructs of the Consolidated Framework for Implementation Research (CFIR) [34] (PROSPERO, CRD42020167697).

##### Findings & lessons learned

 In total, 14 articles were included in this review. The following CFIR constructs were found relevant for implementation: design quality and packaging, adaptability, cost (domain: intervention characteristics); 2): cosmopolitanism, external policy and incentives, patients’ needs and resources (domain: outer setting); implementation climate, readiness for implementation, structural characteristics (domain: inner setting); individual stage of change, knowledge and beliefs about the intervention (domain: characteristics of individuals); engaging, executing, reflecting and evaluating (domain: process). Overall, the CFIR provides a systematic way to identify and classify barriers and facilitators of interventions in the kindergarten and school setting. The strength of the CFIR is that all factors are well defined. However, themes that could not clearly be assigned to any CFIR constructs were identified. Based on this study, we suggested, for example, expanding the CFIR by the determinant “acceptability” to better capture the perceptions of value and perceptions of behaviour change by the target group. In addition, adjustments had to be made about the persons involved, as the CFIR—at the time of the analysis—did not differentiate between implementers and target groups (e.g. parents might be both simultaneously). However, the range of persons involved is covered in more detail in the updated CFIR [45].

For further details, see [26].

#### Participatory approaches are necessary for successful policy implementation evaluations

To gain practical experience with policy implementation evaluation, we conducted three case studies based on different research methods (Case reports 1, 8 and 9) in addition to the previously reported reviews (Case reports 2, 3, 4, 5, 6 and 7). In all case studies, we have applied the Consolidated Framework for Implementation Research (CFIR) [34] to explore implementation determinants of health behaviour-related policies.

For qualitative interviews, there is practical guidance (https://cfirguide.org/guide/app/#/) on how to build a customized interview guide based on the CFIR constructs that are the focus of the evaluation. However, based on our experience (Case report 1), the complete interview guides are extensive and time consuming to implement in practice. Thus, we recommend carefully selecting and focusing on key constructs in terms of barriers and facilitators rather than trying to gather information on all constructs. In addition, questions regarding particular constructs can be formulated more openly than provided in the CFIR guide, asking for descriptions and examples rather than short answers. Open-ended questions give rich information, which can then provide more context and depth to the analysis.

Given the diversity of the CFIR constructs, providing a complete set of quantitative measures seems complicated. However, all quantitative measures mapped to the CFIR are already listed online (https://cfirguide.org/evaluation-design/quantitative-data/), and this list is being updated continuously. Furthermore, in our experience, if there is no suitable questionnaire that can be used or adapted for the research question and setting, the CFIR interview guide tool can help to formulate specific questions on the individual (sub-)constructs (Case report 8).

Co-creation of knowledge on policy implementation, jointly by the target group, implementers, and stakeholders, is more likely to generate practice-oriented outcomes than conducting studies by scientists alone. Following the principles of community-based participatory research, a shared decision-making atmosphere in which everyone plans and decides together may be considered [46]. For this purpose, we followed the Theory of Change approach [47] and engaged different stakeholders in a workshop to map the implementation process of health-related policies in Southern Germany. Theory of Change (ToC) is an outcomes-based approach which describes how an initiative (here a policy) brings about specific outcomes through a logical sequence of intermediate outcomes (e.g., fruit is available in households, children want to eat fruit) that will lead to achieving the goals of the policy (e.g. adequate fruit consumption by children under 16 in households) [47]. Next to the hypothesized outcomes on the causal pathway of policy implementation, the ToC outlines the underlying assumptions and rationales, the interventions required to achieve these outcomes, and the indicators that could be used to measure each outcome on the ToC pathway [47]. A ToC limited to a regional area and involving a wide range of stakeholders may have a greater chance of leading to implementation success as it assures that local resources and capacities are considered. Furthermore, to develop implementation strategies that are inclusive and culturally sensitive, representatives of marginalised groups must be engaged. This way, a common understanding of the problem and potential options will be gained by revealing, exploring and accepting different perspectives [48].

The flexibility in the ToC approach is both an advantage and a disadvantage. On the one hand, ToC can be used to plan or evaluate a wide range of public health policies in almost every setting. On the other hand, ToC from different projects is hardly comparable, as they are very context-specific, and there are differences in how a ToC workshop is conducted (e.g., in terms of moderation, documentation and participants). Several guidance notes exist on developing and using the ToC [47–50]. However, there is no guidance on how to use the ToC in implementation evaluation for public health policies addressing dietary behaviour, physical activity or sedentary behaviour.

#### Case report 8: barriers and facilitators to the adoption of physical activity policies in elementary schools from the perspective of principals: an application of the consolidated framework for implementation research–a cross-sectional study.

##### Objective

To explore barriers and facilitators to adopting physical activity policies in elementary schools in Baden-Wuerttemberg, Germany, from the perspective of school principals.

##### Methods

A quantitative cross-sectional study was conducted between May and June 2021. School principals from elementary and special needs schools (*n* = 2,838) were invited to participate in the study. The online questionnaire used was developed based on the CFIR [34]. It included questions on school characteristics and constructs of the CFIR domain’s inner setting (e.g., tension for change, available resources), characteristics of individuals (knowledge and beliefs about the intervention), and process (engaging stakeholder).

##### Findings & lessons learned

Of 121 participating schools (4% of eligible schools), 49 (41%) reported adopting a policy promoting physical activity. Facilitators for policy adoption included a general willingness among teaching staff, available resources, access to knowledge and information, and the perceived importance of stakeholder engagement (e.g., teachers, school management, parents, students, researchers, politicians). Due to the existing detailed description of the CFIR domains/constructs [34] and the available CFIR Interview guide tool (https://cfirguide.org/guide/app/#/), the CFIR provided good guidance in designing the study and interpreting the results. Overall, the CFIR could be well adapted to the school setting. However, in the absence of further quantitative studies examining the associations between implementation determinants and the adoption of physical activity policies in the school setting, there remains a large knowledge gap in this regard. The development of validated survey instruments for quantitative research based on the CFIR or comparable frameworks could contribute to a better understanding of the underlying mechanisms for the adoption of policies in schools.

For further details, see [27].

#### Case report 9: stakeholder study. Processes, and determinants for the implementation of health-related policies

##### Objective

To identify processes and determinants for implementing policy actions to promote healthy nutrition and physical activity in a federal state in south-west Germany.

##### Methods

In this qualitative study, we conducted two consecutive workshops with members of Municipal Health Conferences to develop a Theory of Change (ToC) for the implementation evaluation of health policies in Baden-Wurttemberg, Germany. In order to categorise identified determinants, they were assigned to the constructs of the CFIR.

##### Findings & lessons learned

 We identified six core processes for the successful implementation of health policies in Baden-Wurttemberg: (1) adequate needs assessment; (2) well-informed decision on the scale and content of health actions; (3) Municipal Health Conferences (MHC) with the ability to act; (4) sufficient funding; (5) proper citizen participation; and (6) reasonable evaluation and dissemination of health actions. These processes were related to different implementation stages (at the beginning, in the course of the process, at the end or permanently) and involved individuals from all levels of policy implementation: political actors (macro: e.g. politicians), socio-political actors (meso: e.g. school principal), and social actors (micro: e.g. teachers). In total, about 100 determinants for policy implementation were mentioned. Mapped to the CFIR, determinants were referring to the implementation process (34%), intervention (policy actions) (24%), inner setting (21%) or outer setting (16%). In the context of health-related policies, individual determinants were rarely mentioned (5%).

Our experience shows that ToC workshops can provide a valuable setting for communication and consultation between stakeholders and researchers. Engaging stakeholders in ToC workshops could gain new insights into local health policy implementation. The findings from the workshops were compiled in ToC-maps that are used to inform further implementation strategies, education of stakeholders and evaluation designs. Especially when systematic data on health policy implementation is unavailable, ToC workshops provide a good starting point by drawing a broad picture of the important processes and determinants from different perspectives while creating a common understanding.

Further strengths of the ToC approach are that additional data sources (e.g., scientific evidence or experiences from single hard-to-reach stakeholders) can be easily included and that other implementation frameworks can complement the ToC. For instance, we coded the assumptions of the ToC Map according to the CFIR constructs to get an overview of how the facilitating and hindering implementation factors are distributed across the different constructs.

### Key recommendations and ten steps instruction for policy implementation evaluation

Based on our experiences with policy implementation evaluation as described above, three key recommendations on the evaluation of policy implementation were derived in a modified Delphi-approach by the authors:

1. Conduct a comprehensive policy implementation evaluation from a multi-level perspective (macro / meso / micro level) throughout the implementation phase. The evaluation design must cover the complexity, dynamics, and interactions of determinants and outcomes.
2. Use implementation frameworks to address implementation processes, determinants, and outcomes by considering the interplay between contextual and equity factors like gender, age, economic status/education/literacy, ethnicity, geographic isolation/distance, and culture. Choosing an appropriate framework depends on many factors, such as the policy to be evaluated and the research question. Therefore, systematic reviews of implementation frameworks [23] and comparison and selection tools [51] are extremely useful in the decision-making process.
3. Engage relevant stakeholders in policy implementation and the evaluation phase. Particular attention should be paid to equity and diversity aspects, e. g. related to gender, cultural background, ethnicity and socioeconomic status. Moreover, the choice of individual stakeholders from different groups (e.g., policy makers, industry, donors, implementers, target group/community) and hierarchical levels should be made by considering the nature of the policy (e.g. nutrition, physical activity) and the context in which the policy is implemented (e.g. public policy, school policy).

**Fig. 1:**
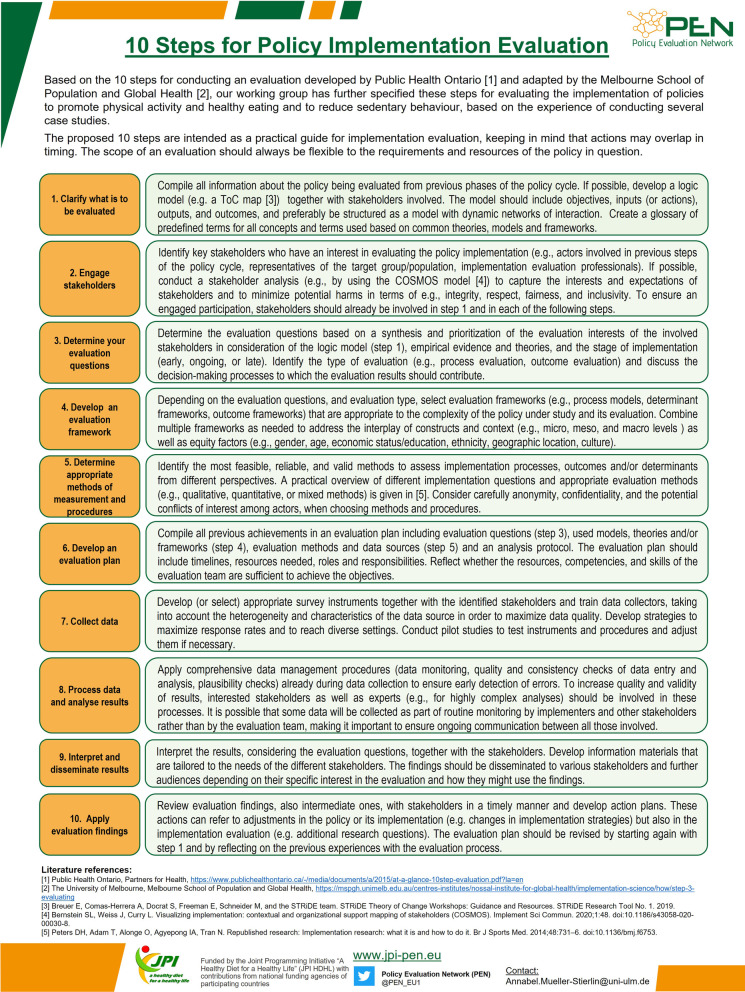
Ten steps for policy implementation evaluation, as initially developed by Public Health Ontario [31], adapted by the Melbourne School of Population and Global Health [32], and specified by the Policy Evaluation Network based on findings and experiences gained in conducting seven reviews and three case studies

The ten steps give a practical guide for evaluating the implementation of health-related interventions and policies developed by Public Health Ontario [31] and adapted by the Melbourne School of Population and Global Health [32]. In light of our experiences (as described in Case reports 1, 2, 3, 4, 5, 6, 7, 8 and 9), we have further specified these steps for evaluating the implementation of policies to promote physical activity and a healthy diet and to reduce sedentary behaviours (see Fig. 1). It remains to be emphasised that the scope of an evaluation must always be flexibly adapted to the requirements and resources of the policy concerned. Nevertheless, the guidelines can serve as a practical orientation for academics and non-academics.

## Discussion

### Strengths and limitations

Given the lack of policy implementation evaluation and related reports or publications and the complexity of the individual cases, it was not feasible for us to provide an overall evidence-based guideline for policy implementation evaluation. However, given these limitations, this interdisciplinary expert group sought to provide appropriately scientifically sound recommendations. To this end, a mixed approach was used. Several systematic searches were conducted in scientific data bases and on stakeholder websites. Representatives of different scientific disciplines and different countries were involved and exchanged expert views on various reviews and case studies over several years. In this way, we combined extensive literature-based evidence with many years of experience in policy implementation evaluation (both practical experience and theoretical knowledge) in this guidance.

The major limitation is the lack of practitioners’ engagement in developing this guidance which also highlights the major challenge for meaningful policy implementation evaluation. Despite the awareness of the importance of and the desire for the involvement of stakeholders in PEN, stakeholder engagement was rather low in general. This may be due to a lack of resources and the fact that our stakeholder engagement studies pursued rather generic questions. Thus, the direct interest of the individual stakeholders was rather low. Overall, improved and adequately financed approaches must be developed and tested to enhance stakeholder engagement in case studies or other research informing the evaluation of policy implementation.

### Implications for research and practice

In future, practical recommendations for identifying stakeholders, their recruitment, and tools for participatory collaboration (e.g. online focus groups or workshops) should be jointly developed or tested in the field of policy implementation evaluation. Furthermore, a comprehensive stakeholder analysis should be conducted, for example using the COSMOS model [52], which provides the opportunity to identify potential stakeholders, understand their relationships, and display their level of support for the proposed implementation and its evaluation. From a holistic perspective, concepts and guidelines should be developed on how to better involve marginalised groups. Participatory approaches should also be compared, and barriers and facilitators of stakeholder engagement should be explored in more detail.

## Conclusions

We found that policy implementation, in general, has rarely been (adequately) evaluated. Based on evidence and practical experience gathered within seven reviews and three case studies, this guidance outlines the approach we would follow for policy implementation evaluation in future projects to ensure that the complexity of policy implementation is sufficiently addressed. Given the current lack of knowledge, even simple monitoring of the implementation processes can contribute significantly to knowledge gain and, thus to the improvement of implementation strategies. For this purpose, we presented three key requirements and ten steps for policy implementation evaluation and highlighted the associated challenges. While our focus was on policies to promote physical activity and healthy diet and to reduce sedentary behaviours, most of the guidance might apply to the implementation evaluation of other health promotion policies, too.

## Supplementary Information

**Additional file 1.**

## Data Availability

The datasets used and/or analysed during the current study available from the corresponding author on reasonable request.
