# Supplementary material for: Good practice recommendations on implementation evaluation for policies targeting diet, physical activity, and sedentary behaviour
Source: BMC Public Health. 2023 Jun 28;23:1259. doi: 10.1186/s12889-023-15775-9 (PMC10308765; doi:10.1186/s12889-023-15775-9)
Supplement: Supplementary file 1 — Additional file 1. [file 12889_2023_15775_MOESM1_ESM.pdf]

Case report/ Reference	Objective	Study design	Data source	Screened/ Included (n)	Participants (n); type *AND* target group	Analysis and applied framework/theory	Key theme / key requirement associated with
**1/[20, 21]**	**What do we know about the actual implementation process of sugar-sweetened beverage taxation and public physical activity policies: Results from two scoping reviews**						
	(1) To examine implementation processes for sugar-sweetened beverage (SBB) taxation<br>(2) To examine implementation processes for physical activitiy (PA) policies	Two scoping reviews	10 databases and grey literature	SBB: 1,248/3;<br>PA: 7,741/10	n/a;<br>SBB: general population, PA: general population, school children	Narrative synthesis;<br>no framework applied	Key theme: scarcity of policy implementation evaluation<br>→ 1st key requirement: comprehensiveness of policy implementation evaluation
**2/[under review]**	**Barriers and facilitators to implementation of the EU School Fruit and Vegetables Scheme: cross country study using the Consolidated Framework for Implementation Research**						
	To identify determinants (barriers and facilitators) to the implementation of the EU School Fruit and Vegetables Scheme (SFVS) from the perspective of country-level government implementers	Qualitative, explorative study	Semi-structured interviews	n/a	29;<br>EU level representatives of the SFVS from ministries of agriculture, health, and education;<br>school children	Qualitative data was coded inductively; the determinants were assigned to the constructs of the CFIR	Key theme: complexity of policy implementation evaluation<br>→ 1st key requirement: comprehensiveness of policy implementation evaluation

<u>Case report/ Reference</u>	Objective	Study design	Data source	Screened/ Included (n)	Participants (n); type *AND* target group	Analysis and applied framework/theory	Key theme / key requirement associated with
**3/[22]**	**Social, economic, political, and geographical context that counts: Meta-review of implementation determinants for policies promoting healthy diet and physical activity**						
	To synthesize the evidence of the context-related implementation determinants of policies targeting physical activity, sedentary behaviour, or healthy diet	Systematic meta-review	9 databases and documentation (evidence-based policy guidelines at national or international level) of 9 major stakeholders from governmental and non-governmental organisations (e.g., European Commission, WHO)*	Peer-reviewed articles: 3,774/25; Stakeholder documents: 52,961/17	n/a; general population, specific settings (school, workplace), populations at risk for obesity	Determinants were coded as belonging to the macro-level (national or country level) and meso/micro-level (community/organizational or individual level); determinants were assigned to the CICI framework	Key theme: complexity of policy implementation evaluation → 1st key requirement: comprehensiveness of policy implementation evaluation → 2nd key requirement: use of frameworks (contextual factors) → 3rd key requirement: stakeholder engagement (contextual factors)

Case report/ Reference	Objective	Study design	Data source	Screened/ Included (n)	Participants (n); type *AND* target group	Analysis and applied framework/theory	Key theme / key requirement associated with
**4/[23]**	**Frameworks for implementation of policies promoting healthy nutrition and physically active lifestyle: systematic review**						
	To provide an overarching synthesis of frameworks guiding the implementation of healthy nutrition, physical activity, and sedentary behaviour policies	Systematic review	9 databases and 8 stakeholder websites (e.g., European Commission, WHO)*	Peer-reviewed articles: 1,578/31<br>Stakeholder documents: 147,887/7	n/a;<br>n/a	Data were coded according to 5 categories: (1) scope of content, (2) level of constructs, (3) types of relationships between constructs, (4) equity factors, (5) direct focus on particular behaviour (e.g., nutrition) vs. discussed application for particular behaviour;<br>No overall framework applied	Key theme: use of frameworks for policy implementation evaluation<br>→ 1st key requirement: comprehensiveness of policy implementation evaluation<br>→ 2nd key requirement: use of frameworks
**5/[24]**	**Acceptability of policies targeting dietary behaviours and physical activity: a systematic review of tools and outcomes**						
	To identify tools used to assess the acceptability of policies targeting physical activity and dietary behaviour and to examine if acceptability differs depending on the policy's and respondents' characteristics	Systematic review	3 databases and Google Scholar	Peer-reviewed articles: 7,236/48	n/a;<br>general population, various populations/settings (e.g., preschool children, school children)	Five categories of information were coded and extracted: (1) types of measures used to gain information on acceptability, (2) levels of acceptability, (3) characteristics of target behaviour, (4) characteristics of policies, (5) characteristics of target respondents: age, sex, country and socioeconomic status, referring to the Outcomes for Implementation Research Framework by Proctor et al. [15]	Key theme: use of frameworks for policy implementation evaluation<br>→ 2nd key requirement: use of frameworks (acceptability)

Case report/ Reference	Objective	Study design	Data source	Screened/ Included (n)	Participants (n); type *AND* target group	Analysis and applied framework/theory	Key theme / key requirement associated with
**6/[25]**	**Meta-review of implementation determinants for policies promoting healthy diet and physically active lifestyle: application of the Consolidated Framework for Implementation Research**						
	To identify determinants that may affect the implementation processes of policies targeting healthy diet, promoting physical activity, and/or reducing sedentary behaviour	Meta-review	9 databases and documentation (evidence-based policy guidelines at national or international level) of 9 major stakeholders from governmental and non-governmental organisations (e.g., European Commission, WHO)*	Peer-reviewed articles: 3,774/25; Stakeholder documents: 52,961/17	n/a; various populations/settings, school children	Data were coded according to 5 categories: (1) policy, (2) implementation, (3) healthy diet policy, (4) physical activity/sedentary behaviour policy, (5) school setting policies; determinants were assigned to the constructs of the CFIR	Key theme: use of frameworks for policy implementation evaluation → 1st key requirement: comprehensiveness of policy implementation evaluation → 2nd key requirement: use of frameworks
**7/[26]**	**Barriers and facilitators to implementation of direct fruit and vegetables provision interventions in kindergartens and schools: a qualitative systematic review applying the consolidated framework for implementation research (CFIR)**						
	To systematically review qualitative results reporting on the determinants (barriers and facilitators) of the implementation of interventions that entail direct provision of fruit and vegetables in kindergarten and school settings	Systematic review	6 databases	Peer-reviewed articles: 5,427/14	n/a; children in kindergartens, primary and secondary schools environments	Determinants were assigned to the constructs of the CFIR	Key theme: use of frameworks for policy implementation evaluation → 1st key requirement: comprehensiveness of policy implementation evaluation → 2nd key requirement: use of frameworks

Case report/ Reference	Objective	Study design	Data source	Screened/ Included (n)	Participants (n); type *AND* target group	Analysis and applied framework/theory	Key theme / key requirement associated with
**8/[27]**	**Barriers and facilitators to the adoption of physical activity policies in elementary schools from the perspective of principals: An application of the consolidated framework for implementation research–A cross-sectional study**						
	To investigate determinants (barriers and facilitators) for the adoption of physical activity policies in elementary schools in Baden-Wuerttemberg, Germany, from the perspective of school principals	Quantitative cross-sectional study	Online survey	n/a	121 primary school headmasters and deputy headmasters	The online questionnaire used was developed based on the CFIR and included questions on school characteristics and constructs of the CFIR domains inner setting, characteristics of individuals, and process; logistic regression analyses were performed to examine associations between PA policy adoption and school characteristics and CFIR determinants	Key theme: participatory approaches for policy implementation evaluation → 2nd key requirement: use of frameworks → 3rd key requirement: stakeholder engagement

Case report/ Reference	Objective	Study design	Data source	Screened/ Included (n)	Participants (n); type *AND* target group	Analysis and applied framework/theory	Key theme / key requirement associated with
**9/[28]**	**Stakeholder study. Processes, barriers, and facilitators for the implementation of health-related policies.**						
	To identify processes and determinants for the implementation of policies to promote healthy nutrition and physical activity being in place in a federal state in south-west Germany	Stakeholder consultation study	Two face-to-face workshops	n/a	7 (first workshop) and 6 (second workshop) participants (members of communal health conferences); general population	Theory of Change approach; determinants were assigned to the constructs of the CFIR	Key theme: participatory approaches for policy implementation evaluation → 2nd key requirement: use of frameworks → 3rd key requirement: stakeholder engagement

Abbreviations: SBB = sugar-sweetened beverage, PA = physical activity, SFVS = EU School Fruit and Vegetables Scheme, CFIR = Consolidated Framework for Implementation Research, CICI = Context and Implementation of Complex Interventions framework

Databases (varies depending on the review): MEDLINE, EMBASE, PsycINFO, Cumulative Index to Nursing and Allied Health Literature (CINAHL), EconLit, Applied Social Sciences Index and Abstracts (ASSIA), Education Resources Information Center (ERIC), PAIS, Scopus, Social Sciences Citation Index (SSCI), Science Citation Index–Expanded (SCI-Expanded), Arts & Humanities Citation Index (A&HCI), Book Citation Index–Science (BKCI-S), Book Citation Index–Social Sciences & Humanities (BKCI-SSH), PsycARTICLES, Nursing/Academic Edition, Academic Search Ultimate, AGRICOLA, Cochrane Database of Systematic Reviews (CDSR), Database of Abstracts of Reviews of Effects (DARE), Science Direct, PubMed, Web of Science

Grey literature (varies depending on the review): OpenGrey, ThinkTank, and BASE, Google Scholar

*Stakeholder websites: National Institute for Health and Care Excellence (United Kingdom), the European Commission (e.g., Consumers, Health, Agriculture and Food Executive Agency), World Health Organization, Regional Office for Europe, Centers for Disease Control and Prevention (USA), National Academy of Medicine (USA), Australian Department of Health, National Health and Medical Research Council (Australia), Organization for Economic Co-operation and Development, and Food and Agriculture Organization of the United Nations

Brief description of frameworks used:

(1) **Consolidated Framework for Implementation Research (CFIR):** The CFIR is a determinant framework that provides a systematic guide for assessing determinants for the implementation of health-related interventions. It can be considered as "meta-theoretical" as it was developed by synthesizing constructs from various existing implementation theories. The framework lists 26 key determinants, which are grouped into five domains: (1) intervention characteristics (2) inner setting (3) outer setting (4) individuals involved, and (5) implementation processes. For example, the inner setting specifies any characteristics of the implementing organization (e.g. school), through various constructs (e.g., structural characteristics, communications, culture, tension for change). The outer setting is used to capture macro-level factors (e.g., external policies) that emanate from outside the inner setting [34].

(2) **Context and Implementation of Complex Interventions (CICI) framework:** The CICI framework helps to simplify and structure complexity to advance our understanding of whether and how interventions work. It consists of the three dimensions context, implementation and setting. The dimension context comprises the seven domains geographical, epidemiological, socio-cultural, socio-economic, ethical, legal, political context. Context factors might be represented at the macro (e.g., country-level characteristics), meso, and micro levels (e.g., characteristics of the target organizations, target individuals). For example, the economic context consists of economic resources at the macro level (e.g., national funds) and meso/micro level factors such as access to the economic resources of individuals or organizations [12].

(3) **Theory of Change (ToC):** The ToC is a participative process that is used in the development, implementation and evaluation of projects and complex interventions by engaging (local) stakeholders in ToC workshops [49]. A ToC is a theory of how and why an initiative works [52]. With a backward-mapping approach, the ToC aims to show the pathways through which an intervention is expected to achieve its objectives while specifying the factors influencing these pathways. The results of ToC workshops usually consist of two components, a visual (ToC map) and a narrative representation that contains information about the context in which the intervention is implemented (e.g. social, political and environmental conditions), the actors involved, the change process and the underlying assumptions.
